# Emotional intelligence as a predictor of identified regulation, introjected regulation, and external regulation in athletes

**DOI:** 10.3389/fpsyg.2022.1003596

**Published:** 2022-10-14

**Authors:** Isabel Mercader-Rubio, Nieves Gutiérrez Ángel, Nieves Fátima Oropesa Ruiz, José Juan Carrión-Martínez

**Affiliations:** ^1^Department of Psychology, Universidad de Almería, Almería, Spain; ^2^Department of Education, Universidad de Almería, Almería, Spain

**Keywords:** motivation, emotional intelligence, athletes, regulation, students

## Abstract

Self-determination theory (SDT) considers motivation as a multidimensional phenomenon, with different levels of intensity, purposes, intentions, wills and autonomies. It distinguishes between intrinsic motivation (IM), extrinsic motivation (EM) and amotivation (AM). In this paper, we are going to focus on extrinsic motivation, which is related to those tasks that the subject performs without having a purpose in themselves, and which is composed of identified regulation, introjected regulation and external regulation. The aim of this research is to analyse the relationship between them and emotional intelligence in 165 students with university degrees related to Physical Activity and Sport Sciences. The main findings of this work lie mainly in the demonstration of the fact that emotional intelligence is a predictor of identified regulation, introjected regulation and external regulation.

## Introduction

The purpose of sport is to show or improve physical and psychological condition, to develop social relations and to obtain better results (Unisport, 1992). Therefore, it is a widely recognised field of research to investigate different variables such as the influence of motivation (León et al., 2017). Motivation has important implications for athletes (Irwin and Feltz, 2016), as it is considered an essential component for obtaining commitment and adherence to sports practice, consolidating itself as the most important and immediate psychological determinant in the behaviour of the subject (Iso-Ahola and Clair, 2000).

In line with these ideas, when we talk about motivation, in general, we refer to the predisposition of a subject to perform a task with a certain intention (Palmero, 2005; Vallejo-Reyes et al., 2018). The study of motivation and behaviour in physical activity has been approached from two theoretical frameworks: achievement goal theory (Nicholls, 1989) and self-determination theory (Deci and Ryan, 1985; Ryan and Deci, 2000). In the scientific literature, several review papers on achievement goal theory (Braithwaite et al., 2011) and self-determination theory (Curran and Standage, 2017; Sun et al., 2017; Van den Berghe, et al., 2014) can be found.

In this sense, one of the main psychological theories on this construct is Self-Determination Theory (SDT). This theory considers self-determination as a multidimensional phenomenon, with different levels of intensity, purposes, intentions, wills and autonomies (Deci and Ryan, 1985). Therefore, this theory distinguishes between intrinsic motivation (IM), extrinsic motivation (EM) and amotivation (AM).

There are several investigations carried out in the field of sport under the prism of this theory (Li and Harmer, 1996; Martens and Weber, 2002; Guzmán et al., 2006; Balaguer et al., 2007; Mallett et al., 2007; Martin-Albó et al., 2007; López, 2010; Pelletier et al., 2013; Guevara et al., 2015; León et al., 2017; Muñoz, 2021; Vallejo-Reyes et al., 2018) which aim to inquire about the motives that lead the athlete to both initiate and maintain a behaviour (Trigueros-Ramos, et al., 2019; Navarro-Patón et al., 2020; Mercader-Rubio et al., 2022; Mossman et al., 2022).

However, in this paper, we are going to focus on extrinsic motivation, which is related to those tasks that the subject performs without possessing a purpose in themselves (Vallejo-Reyes et al., 2018). Therefore, taken to the field of sport, we speak of that type of motivation that leads the athlete to the task that is a means to other ends, where the locus of causality becomes external (Balaguer et al., 2007).

In addition, within extrinsic motivation, we find four levels, which correspond to different types of regulation (Deci and Ryan, 1985; Vallejo-Reyes et al., 2018). Integrated Regulation (INTEG): It corresponds to the highest level of self-determination and occurs when the cognition of motivation is related and in accordance with the subject’s own self-concept and values (Vallerand and Bissonnete, 1992). It represents the highest level of self-determination. Identified Regulation (IDR): It corresponds to the fact that the cognition of motivation is considered relevant, despite the fact that the task is exercised largely for extrinsic reasons. In this case, we therefore refer to an internally regulated behaviour. And it corresponds to medium levels of self-determination. Introjected Regulation (INTY): It corresponds to the mental image of external contingencies. In this type, we find that the subject either possesses the behaviours fully internalised, but tends to exercise them by imposition or guilt. It corresponds to low levels of self-determination. External Regulation (EXT): It corresponds to those actions that are directly controlled by external stimuli and corresponds to low levels of self-determination.

However, the creators of the SMS/EMD instrument developed and validated an instrument in which there are some modifications to the different types of motivation proposed in the self-determination theory (Balaguer et al., 2007), in which extrinsic motivation is composed of three types: identified regulation, introjected regulation and external regulation. These are the ones that have been taken in this work.

In short, in the field of sport, motivation is one of the most studied variables as it is closely related to those motives that lead the athlete to start, maintain and abandon sport practice (Guillén, 2007; Muñoz, 2021).

Another of the constructs that has been widely studied in sport psychology is emotional intelligence (Ribeiro et al., 2018; Cowden, 2020). A large number of studies on this construct have selected athletes as a sample in order to clarify the relevance and importance of emotional intelligence in the field of sport. The results provided by them indicate that those athletes who obtain higher scores in the levels of emotional intelligence have greater effectiveness at the competitive level (Lott and Truner, 2018), or in the different dimensions of self-concept (Vaughan and Laborde, 2017). And several studies have shown a direct and positive correlation between emotional intelligence and motivation in the field of sport (Castro-Sánchez et al., 2018, 2019; Gallegos and López, 2020; Méndez-Giménez et al., 2021; Mercader-Rubio et al., 2022), although there is not much agreement on the differences established between sexes in this regard (Luna et al., 2019; Sánchez et al., 2021; Tinkler et al., 2021).

When talking about emotional intelligence, therefore, we have to highlight the fact that two models coexist to express and understand the concept of emotional intelligence: mixed models and ability models. However, this research specifically focuses on ability models (Mayer and Salovey, 1997), which are composed of sections or branches: Emotional perception: refers to the ability to match and examine one’s own and others’ feelings. This involves, therefore, attention to and recognition of the expression of different symbols, emotions and clarity of feeling. Emotional understanding: this involves recognising, cataloguing and exploring emotions in retrospect, both one’s own and those of others. Emotional regulation: this is about apprehending, comparing and deliberating about emotions, both interpersonally and intrapersonally.

Thus, the results show that high levels of emotional intelligence correlate directly and positively with motivation (Castro-Sánchez et al., 2019; Gallegos and López, 2020; Méndez-Giménez et al., 2021; Mercader-Rubio et al., 2022). This leads us to create the main research question for this work, what type of relationship exists between emotional intelligence and each of its dimensions and identified regulation (FD), introjected regulation (FT) and external regulation (FC)?

Therefore, this research aims to analyse the Self-Determination Theory (SDT), specifically in extrinsic motivation (EM) and specifically in identified regulation, introjected regulation and external regulation, and their relationship with emotional intelligence.

Under the formulation of the following hypotheses:

*H1.* There is a direct and positive relationship between emotional attention and identified regulation, introjected regulation, and external regulation.

*H2. H1:* There is a direct and positive relationship between emotional clarity and identified regulation, introjected regulation, and external regulation.

*H3. H1:* There is a direct and positive relationship between emotional regulation and identified regulation, introjected regulation, and external regulation.

## Materials and methods

The methodology used corresponds to an *ex post facto*, retrospective and comparative design, since the dimensions of emotional intelligence are compared with other types of variables, in this case with identified regulation, introjected regulation and external regulation.

### Participants

The total sample consisted of 165 undergraduate and master’s degree students related to Physical Activity and Sport Sciences. Their mean age was 20.33 years, with a standard deviation SD = 3.44. With regard to sex, 70.9% (*n* = 117) were men and 27.9% (*n* = 46) were women. The sample size was determined by the number of students who, with prior information and consent, decided to participate in the study. All participants completed an official informed consent form from the University of Almeria (Spain) and were informed of the data protection protocol (Table 1).

**Table 1:** Description of the sample according to age and sex.

	Females	Males	Total	Menos de 25	Mayores de 25
First course	18 (39.1)	68 (58.1)	86	86 (97.8%)	2 (2.2%)
Second course	16(34.8)	23 (19,7)	39	38 (97.4)	1 (2.6%)
Third year	6 (13%)	14 (12%)	20	20 (100%)	0
Total	40	105	145		
Master’s degree	6 (13%)	12(10.3)	18	4 (22.3%)	17 (77.7%)
Total	46	117	163		

Inclusion criteria took into account that each participant was an official student of the course in which the questionnaires were administered, that they signed the informed consent form (official model of the University of Almeria) and that they were of legal age.

As exclusion criteria, we eliminated those questionnaires that were not 100% completed, or that lacked socio-demographic information. In addition, the questionnaires were collected on paper, so another exclusion criterion was to check that there was no randomness in the answers or that the answers formed drawings.

The type of sampling used was simple random, as the questionnaire was given to all the students who attended class that day, with the prior agreement and permission of the teacher responsible for the subject.

**Figure 1:**
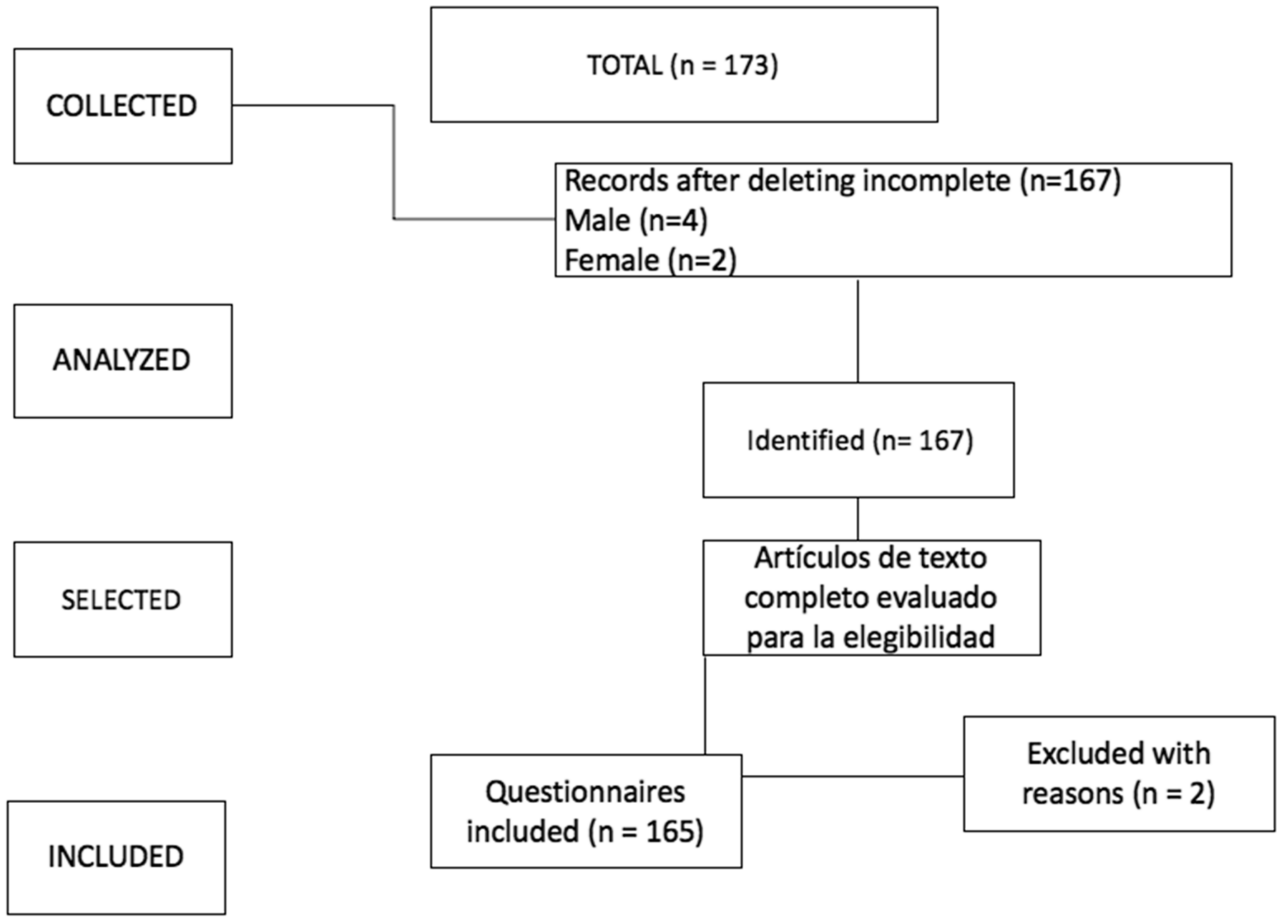
Flowchart of the selection procedure.

The sample size was determined according to the number of students who, with prior information and consent, decided to participate in the study. The questionnaire was administered to all four undergraduate courses in physical activity and sport sciences. The questionnaire was administered to students of the master’s degree in teaching (specialisation in physical education) and the master’s degree in sport science research (Figure 1).

### Instruments

Two instruments were used in this work:

The TMMS-24 (Fernández-Berrocal et al., 2004). It corresponds to a self-report that measures self-perceived emotional intelligence, i.e., the subject’s knowledge of his or her own emotional capacities: attention to feelings, emotional clarity and emotion regulation, using a Likert-type scale (1–5). A Cronbach’s alpha = 0.84 was obtained for this work. In addition, it shows high reliability (Cronbach’s alpha) for each dimension (perception, α = 0.90; clarity, α = 0.90; regulation α = 0.86) and adequate test–retest reliability: perception = 0.60; understanding = 0.70 and regulation = 0.83.

And, the Sport Motivation Scale (SMS/EMD; Pelletier et al., 1995; Balaguer et al., 2007). It is a Likert-type scale (1–7) that measures motivation through seven subscales: (1) intrinsic motivation to experience stimulation; (2) intrinsic motivation to achieve things; (3) intrinsic motivation towards knowledge; (4) identified regulation; (5) introjected regulation; (6) external regulation and (7) amotivation. In this research, we only took data from subscales 4-5-6. For this work, we obtained a Cronbach’s alpha = 0.73. In addition, it shows high reliability (Cronbach’s alpha) for each subscale (identified regulation, α = 0.74; introjected regulation, α = 0.73 and external regulation α = 0.79).

To calculate the variance in the total scores, the hierarchical omega (ωH; Zinbarg et al., 2006) was computed. Magnitudes ≥ 0.70 indicate the presence of a unidimensional structure (Reise et al., 2013). In addition, the hierarchical omega (ωHs; Zinbarg et al., 2006) was calculated to estimate the reliability of the scores. Where ωHs values ≥0.30 are considered as significant (Smits et al., 2020). The common variance explained (ECV; Stucky and Edelen, 2015) was also calculated. Values below 0.70 indicate multidimensionality, whereas values above 0.85 are considered unidimensional.

The percentage of untainted correlations (PUC) score was also taken into account. If ECV and PUC are >0.70, or if PUC has a high coefficient (>0.80), and ECV and ωH magnitudes of >0.60 and ≥0.70, respectively, these are favourable indications of unidimensionality.

At the item level, the ECV-I (Stucky and Edelen, 2015) was calculated, which indicates the percentage of the variance of each item explained by the FG. Values ≥ 0.80 indicate a significant influence of the FG (Stucky and Edelen, 2015).

On the other hand, reliability was estimated using the omega coefficient (ω; McDonald, 1999), which measures how a latent variable is represented by a set of items. Values > 0.70 suggest a latent variable adequately defined by its indicators (Hancock and Mueller, 2005).

### Data analysis

The data analyses used in this study were descriptive statistics (mean, standard deviation and bivariate correlations), reliability analysis and structural equation modelling (SEM) to test the relationships established in the hypothesised model.

Specifically, we applied Joreskog’s test for the analysis of covariance structure (Jöreskog, 1970, 1973) to a multiple cause indicator (MIMIC). The rationale for using this test is that we are dealing with a situation where the latent variable is defined as a composite of a set of measures, i.e. the measures produce the constructs. This is called formative indicators. Structural equation systems such as MIMIC solve this.

To accept or reject the proposed model, a set of suitable indices were taken into account (Hu and Bentler, 1999): TLI (Tucker—Lewis index), SRMR (standardised root mean square residual) and RMSEA (root mean square error of approximation). Thus, the appropriate indices are as follows: TLI values above 0.95; SRMR values below 0.06 and RMSEA values below 0.08. These analyses were carried out with SPSS software (version 26) and R statistical analysis programme (version 2015) and the analysis modules belonging to the “lavaan” package.

## Results

The data analysis shows the following bivariate correlations where the relationships between each of the dimensions of emotional intelligence (AE/CE/RE) and their relationship with identified regulation (FD), introjected regulation (FT) and external regulation (FC) are shown in Table 2, where the correlations between the positive and reciprocal study variables are evident.

**Table 2:** Preliminary analyses.

	1	2	3	4	5	6
1. FACTOR 2		0.639^**^	0.328	0.257^**^	0.222^**^	0.215^**^
2. FACTOR 3			0.481^**^	0.264^**^	0.115	0.081
3. FACTOR 4				0.178^*^	0.–007	0.138
4. AE					0.127	0.263^**^
5. CE						0.508^**^
6. RE						

* *p* < 0.05;

** *p* < 0.01.

**Figure 2:**
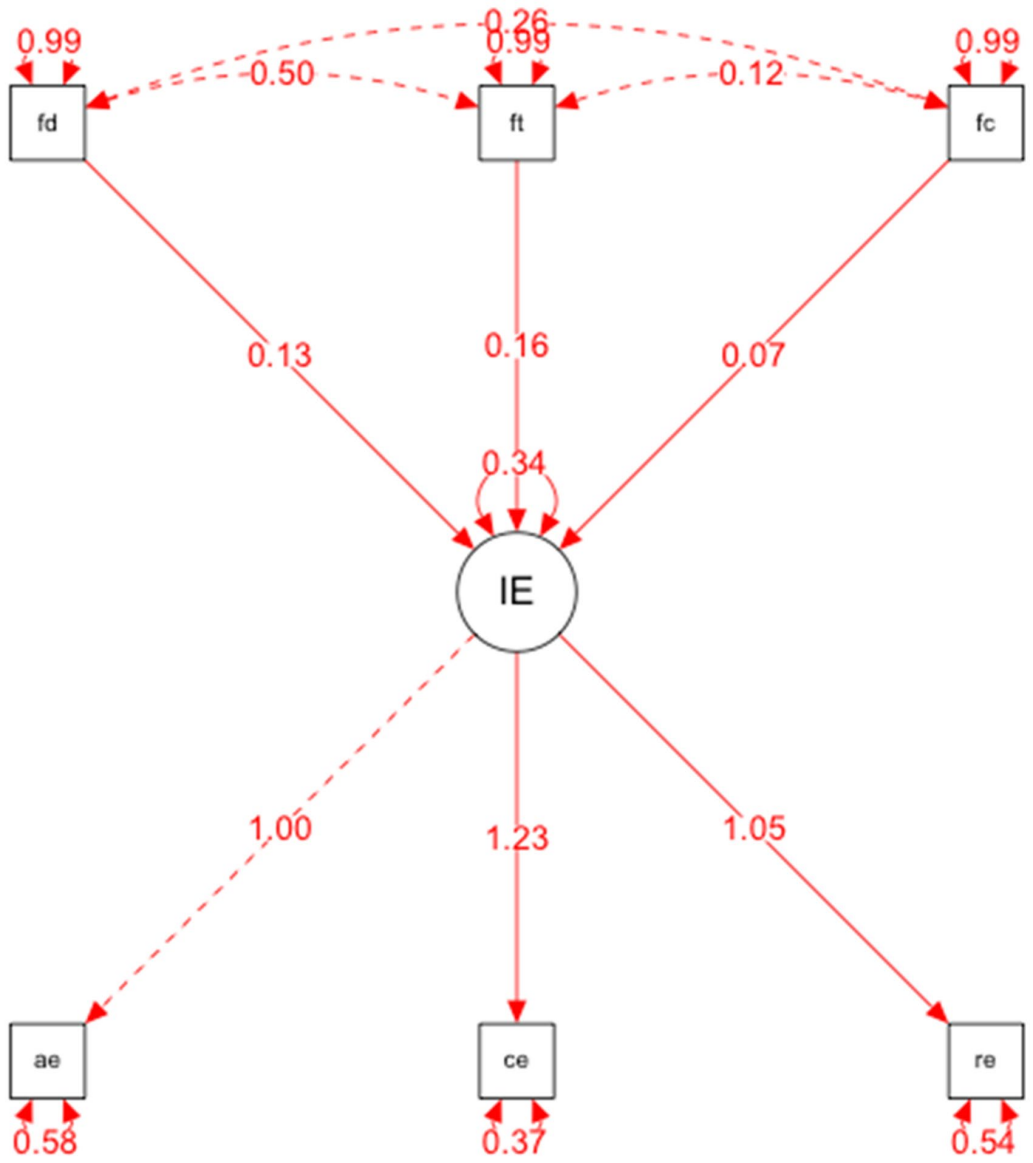
Structural equational modelling.

On the other hand, as for the structural equation models (SEM) to test the relationships established in the hypothetical model, the hypothetical model of predictive relationships (Figure 2) has shown that the overall fit indices (evaluating the model in general) were adequate: *p* < 0.001, RMSEA = 0.038 and GFI = 0.971.

As well as the incremental or comparative fit indices (comparing the proposed model with the model of independence or absence of relationship between the variables) were NNFI = 0.917; TLI = 0.963; CFI = 0.982 and IFI = 0.983.

And the parsimony indices (assessing the quality of the model fit in terms of the number of coefficients estimated to achieve this level of fit) were AGFI = 0.900.In addition, two further indicators were calculated: Composite Reliability (CR), which indicates acceptable reliability (Heinzl et al., 2011), and the AVE (Average Variance Extracted) index, which measures the variance captured by a construct relative to the other constructs in the model (Fornell and Larcker, 1981).

The relationships established in the structural equation model are specified below and their relationship with identified regulation (FD), introjected regulation (FT) and external regulation (FC).

1. Emotional intelligence and factor two: Identified regulation was positively correlated (=0.13, *p* < 0.001). This explains that, in this case, emotional intelligence is a predictor of introjected regulation; therefore, the presence of this variable explains the existence of the other variable.
2. Emotional intelligence and factor three: Introjected regulation was positively related (=0.16, *p* < 0.001). The results show that emotional intelligence also predicts introjected regulation in an explanatory way.
3. Emotional intelligence and factor four: External regulation was positively correlated (=0.07, *p* < 0.001). This explains that, in this case, emotional intelligence is a predictor of external regulation; therefore, the presence of this variable explains the existence of the other variable.

Therefore, based on these results, we can affirm that emotional intelligence is a predictor of extrinsic motivation.

## Discussion and conclusion

The aim of this research has been to analyse from the Self-Determination Theory (SDT), specifically in extrinsic motivation (EM), and specifically in identified regulation, introjected regulation and external regulation, and its relationship with emotional intelligence: attention, clarity and emotional regulation. Therefore, the research question is, what kind of relationship exists between emotional intelligence and each of its dimensions and identified regulation (FD), introjected regulation (FT) and external regulation (FC)? This is answered with the results provided by this work. Therefore, hypotheses 1, 2, and 3 are fulfilled.

The main findings of this work lie mainly in the demonstration of the fact that emotional intelligence is a predictor of identified regulation, introjected regulation and external regulation, from which we deduce the direct and positive correlation between levels of emotional intelligence and levels of self-determination (Méndez-Giménez et al., 2017, 2020, 2021).

Furthermore, this work demonstrates the close relationship between motivation and emotional intelligence in the field of sport, as previous research has already done (Castro-Sánchez et al., 2018, 2019; Gallegos and López, 2020; Méndez-Giménez et al., 2021; Mercader-Rubio et al., 2022).

In such a way, taking these results as an axis, within the sports field, they serve as reference data to take into account and understand the pre-eminence of an intervention that is not only cognitive, but also psychological and emotional, in relation to the competencies of the athlete in the context in which he or she performs (Calero and González, 2014). This will allow full decision-making when implementing different actions that effectively implement the results (Iglesias et al., 2012) through the management of different psychological variables considered key in this area (Calero et al., 2008).

In this sense, it is worth highlighting the relevance of the presence of the sports psychologist and emotional training, since it has been demonstrated that working on and improving emotional intelligence has an impact on the athlete with greater efficiency at a competitive level (Lott and Truner, 2018), or even on their own self-concept (Vaughan and Laborde, 2017).

The main conclusions of this study lead us to stress the idea of the importance of the sports psychologist and the study of those psychological variables that allow the athlete to obtain a greater and better performance. In this particular case, this work shows how emotional training is a good tool for improving the athlete’s abilities and motivation.

We must not forget to be cautious with these results due to the size of the sample. Therefore, we believe that it would be useful to replicate this research with larger samples in order to be able to contrast these observations, as this is one of the current limitations of the work.

Future lines of research will aim to analyse whether there are discrepancies according to the type of sport practised. Thus, future studies will try to find out the contrasts according to the degree of professionalisation of each sport practised by the future participants.

## Data availability statement

The raw data supporting the conclusions of this article will be made available by the authors, without undue reservation.

## Ethics statement

The studies involving human participants were reviewed and approved by UALBIO2022/035. The patients/participants provided their written informed consent to participate in this study.

## Author contributions

IM-R, JC-M, and NÁ: conceptualization. NÁ: methodology. JC-M: software. IM-R, NR, NÁ, and JC-M: research analysis. NR: data curation. NÁ and NR: original drafting—drafting. NÁ and JC-M: drafting—revising and editing. IM-R: supervision. All authors contributed to the article and approved the submitted version.

## Conflict of interest

The authors declare that the research was conducted in the absence of any commercial or financial relationships that could be construed as a potential conflict of interest.

## Publisher’s note

All claims expressed in this article are solely those of the authors and do not necessarily represent those of their affiliated organizations, or those of the publisher, the editors and the reviewers. Any product that may be evaluated in this article, or claim that may be made by its manufacturer, is not guaranteed or endorsed by the publisher.
